# The New Lunacy Laws

**Published:** 1890-07-12

**Authors:** 


### THE NEW LUNACY LAWS.

WITH the first day of May came into operation the new Lunacy Act, and many changes and alterations (we wish we could say, in all cases, improvements) were introduced into our ways of dealing with the insane.

No person can now be received as a private patient into any asylum unless under a reception order made by a judicial authority. The order must, as heretofore, be accompanied by two medical certificates, and these certificates must be on separate sheets of paper, with what object is not easily seen. The petition asking for the order is, if possible, to be signed by the husband or wife or a relative. After admission the patient must be visited once in six months by the petitioner or by some one specially appointed by him. The judicial authority who makes the order is allowed seven days to consider the matter, so that seven precious days may be wasted. The judicial authority may make an order forthwith, or he may take another fourteen days to get information, or he may dismiss the petition altogether. In other words, a magistrate, who has probably no knowledge of medicine, may set aside the opinion of two medical men.

In case of dismissal of petition, and if another petition be presented, the person presenting it must mention the preceding petition or be guilty of misdemeanour. If a private patient be received into an asylum without having been seen by a judicial authority, he may claim the right to be taken before, or visited by, some judicial authority other than the one who signed the order unless the medical officer certify that such a proceeding would be injurious.

Private patients may be received under an urgency order accompanied by a medical certificate, and the order should be made by husband, or wife, or relative. The urgency order remains in force for seven days, or if a petition for a reception order is pending, then until the petition is finally disposed of. It does not seem very clear who is to inform the asylum medical officer whether the petition is pending.

Information may be given on oath to a judicial authority by a constable, overseer, relieving officer, or other person, that a lunatic who is not a pauper and not wandering at large, and not under proper care and control, or is cruelly treated or neglected, and the judicial authority may himself visit the alleged lunatic, and he shall whether or not authorise two medical practitioners to visit and examine and to certify their opinion as to his mental state, and the justice shall then proceed in the same manner as if a petition had been presented. The definition of a pauper under the Act is very clear: so clear that one is tempted to believe it is an interpolation.

The medical certificates accompanying a petition or an urgency order must not be signed by the petitioner, nor by any relative or connection of his. It is properly provided that one of the medical certificates must wherever practicable be by the patient's usual medical attendant. No patient can be received into an asylum if any certificate has been signed by anyone interested in the asylum, and neither of the persons signing the medical certificates shall be "father or father-in-law, mother or mother-in-law, son or son-in-law, daughter or daughter-in-law, brother or brother-in-law, sister or sister-in-law, or the partner or assistant of the other of them." But apparently they may be husband and wife, or father and stepson, or father and stepdaughter.

Reception orders are now of limited duration. They all expire at the end of one year from their dates, unless the medical officer shall certify that the patient is still insane and a proper person to be detained, after which the order shall be valid for two years, then for three years, and five years, and successive periods of five years. A report on the mental state of a private patient shall be sent to the Commissioners in Lunacy at the expiration of one month from admission; this to be in addition to the one sent within seven days.

Mechanical restraint has now a direct legal sanction which it never had before in England; but it cannot be used unless it is necessary for medical or surgical purposes, or to prevent the lunatic from injuring himself or others. A record is to be sent to the Commissioners in Lunacy every quarter. The *necessity* for legalising restraint does not seem apparent to those conversant with the English system, and the *expediency* of legalising it is more than doubtful.

Letters must be forwarded to the Lord Chancellor, or to any Judge in Lunacy, or Secretary of State or Commissioner, or to the person who signed the reception order, or to any of the visiting committee. This merely legalises an almost invariable asylum rule; but letters written to other persons by private patients and not forwarded need not now be laid before the Commissioners. Any patient may now be examined, if the Commissioners consent, by two medical practitioners, and if after a second examination they certify to the Commissioners that the patient ought to be discharged, the Commissioners may order the discharge of such patient. The old "fourteen days'" clause relating to the capture of escaped patients is retained, and a dangerous lunatic may remain at large if he be sufficiently cunning to conceal himself for a fortnight.

All the old private asylums are to be allowed to remain, but no new ones are to be licensed—a clause which must warmly commend itself to all owners of private asylums, but which is hardly calculated to please the Lunacy Law Reform agitators.

There is a new definition of the word "relative." It no longer means "husband" or "wife." There is a useful provision whereby lunatics may have their property taken care of and yet may remain at large themselves. Medical men cannot have vexatious actions brought against them for signing certificates provided they acted in good faith.

County Councils may now build asylums for private patients. Unfortunately this is merely a permissive clause, hence it is much to be feared that it will be inoperative. An "officiating clergyman" can no longer sign an order for the reception of a pauper patient. The pensions' clause relating to county asylum officials is left in the same objectionable form as in the old Acts. There is no limit placed to the sizes of county asylums, and it is much to be feared that they will still further increase their already unwieldy bulk. There is no provision for the transfer of Irish and Scotch lunatics to their own countries. We note with pleasure that two private patients may now be kept in the same private house.

The worst provision is that which requires the interference of a Judicial Authority in the medical affairs of a private individual, and we doubt whether Englishmen will calmly submit to this.

We would ask, What has the lunatic gained by the New Act? Perhaps some lunatic will answer.

